# Collectanea

**Published:** 1826-08

**Authors:** 


### COLLECTANEA.

Floriferus, ut apes, in saltibus omnia libant,
Omnia nos, itidem, depascimur aurea dicta.

### PHYSIOLOGY.

### *Digestion.*

Dr. Smith, of Philadelphia, has arrived at the following conclusions, as the result of numerous experiments on digestion:

1st. That there is no fluid secreted by the stomach, possessing the remarkable chemical properties attributed to the gastric juice, and consequently that chemical solution does not take place in that organ.

2d. That there is a neuro-electric, or an electro-animal principle, conveyed by the nerves of the stomach, and made to act upon its contents through the medium of its saline secretions, which possess sufficient animal properties to facilitate its transmission, and to aid the effect.

3d. That the aliment in the stomach thus undergoes what may be termed an animal decomposition, and, after being reduced to its elements, is recombined by the principle which controls the composition of animal matter, though not probably endued with any properties of life, till it enters the circulation.

4th. That a very considerable portion of the aliment is absorbed by the capillary veins of the stomach and intestines, and consequently conveyed through the vena portæ and the capillary system of the liver, in which route it undergoes still more important changes, and becomes endued with some of the vital properties of the blood.

5th. That another portion is taken up by the lacteal absorbents, by which it is converted into chyle.

It may, perhaps, by some be thought presumptuous in me, (says Dr. S.) thus to attempt the refutation of opinions substantiated by numerous experiments. If any apology be necessary for the freedom which I have used in this discussion, I would observe, that, whenever a fact is brought forward, for whatever purpose it may have been employed by the discoverer, it at once becomes the property of the literary community, and may be used for any logical purposes. It is generally the case that experimenters, intent upon some preconceived hypothesis, take but a partial view of the relations which their results bear to the rest of the science. Although they contribute to the completion of the structure, by bringing together the materials which nature has dispersed through her works, the scientific arrangement of them is usually reserved for other hands. (*American Medical Rev.* No. 1.)

---

### PATHOLOGY.

*Observations on Cataract.* Read to the French Institute, by L. F. Gondret, m.d.

That morbid change in the organ of sight, which consists in opacity of the crystalline lens or its capsule, and to which the name of cataract has been applied, belongs, both by its situation and by the causes which give rise to it, to the class of internal disorders. The remarkable want of success which has hitherto attended all attempts to remove it by medical treatment, has, however, universally transferred the management of it to the hands of the surgeon. It must be acknowledged, that the operations for the cure of this affection have, of late, attained a high degree of perfection, and that a proportionate number of striking cures has been the result. It could be wished, notwithstanding, that a combined practice might be directed against this malady, which frequently becomes the cause of one of the most trying infirmities of age, and occasionally is met with in the earliest periods of life.

The author, in treating epilepsy and mania by cauterisation of the head, had had occasion to notice a remarkable improvement in amaurosis and cataract, in cases in which they happened to coexist with the affection which formed the principal object of attention. The observation appeared perfectly admissible, for there is no more difficulty in conceiving the removal of the physical cause of amaurosis or cataract, than that of mania or epilepsy.

The result of the author's experience with respect to amaurosis, was laid before the Institute in a former paper, and the attention which that received encouraged him in the prosecution of the researches detailed in the communication now before us.

In seeking to apply to cataract the treatment which he had adopted in chronic affections of the head, and in gutta serena, Dr. Gondret soon found that his facilities for experiment were much less considerable. The cases of amaurosis which presented themselves were numerous, because this affection is frequently abandoned as hopeless; but, operation being the generally received method of treatment in cataract, it was not till after long waiting that a few almost accidental opportunities occurred for the trial of a practice, which he conceived equally applicable to this as to other affections of the eyes or brain.

He carefully noticed the facts as they occurred, and waited until he had accumulated a number of analogous results, before he considered himself warranted in drawing any thing like a general conclusion.

The seat of the derangement was particularly favourable to these researches. The existence of cataract becomes obvious long before it has arrived at an advanced stage, and it is well known that surgeons generally wait to perform the operation for its cure, until the opacity of the lens and loss of vision are complete. Other internal affections are materially different. A direct examination cannot be made, and the diagnosis is necessarily less positive, being rather the result of induction than of the immediate evidence of the senses.

As a guard against the possibility of his too hasty admission of facts in support of his own views, Dr. Gondret made his observations in conjunction with several of his enlightened professional brethren, who would not have failed to warn him, had he been falling into error.

He took particular care not to be misled, by mistaking an apparent for a real cure, as is often unavoidably the case, where the practitioner too soon loses sight of his patient. He saw the necessity of continuing the observations as long as possible, and did so for months, and even years. We subjoin one case in illustration:

M. Pepin, aged fifty-nine, of a good constitution, but of rather a nervous temperament. After a forced march, in the year 1816, he was attacked with severe fever, accompanied by intense headache and delirium, and followed by a general swelling of the limbs. During his convalescence, he perceived, for the first time, a black speck, which seemed to dance between his right eye and the objects which he looked at. Six months after, a cloudiness, the precursor of cataract, presented itself in the left eye. In the winter of 1819-20, he experienced, during the space of a month, the most exquisite pain in the right temple: this was succeeded by inflammation of the left eye, which was relieved by leeches. In January 1822, the pains in the right temple returned with great severity, but ceased by the use of an opiate pill. In April, Pepin consulted Dr. Gondret. At this time he could see nothing with the left eye, in which the cataract had now existed four years. A grey spot in the centre of the crystalline lens indicated the commencement of cataract in the right eye also. He was unable to read, for more than a short time, without fatigue. The floating spots which he perceived with the right eye were become more numerous, the pupils were rather dilated, and the irides almost fixed.

Dr. G. considers that the malady with which Pepin had been attacked six years previously, as already mentioned, was the principal cause of the derangement of the visual organs: that the brain, in the first instance, becoming affected, an extension took place, producing—

1st. Numerous filaments, and approach to opacity of the crystalline, with feebleness of vision in the right eye.

2dly. Complete cataract of the left eye.

3dly. Pains in the right temple.

4thly. Inflammation of the right eye.

Continued application at the desk had, doubtless, favoured the progress of the complaint.

Guided by experience of the excellent effects of a local treatment in some of the several affections of the brain, even when congenital, Dr. G. was induced to propose to his patient cauterisation of the sinciput, as offering the most favourable chance of relief. He hoped to arrest the progress of the cataract in the right eye, and to render the parts more susceptible to the influence of other agents, which, whether local or general, might favour the restoration of the organ to its natural state.

On the 4th of April, the cauterisation was performed with ammoniacal pomade.

On the 22d of May, the cloud, which had seemed to cover the right crystalline was become less; vision was a little improved, but filaments still remained. An electric current, produced by a voltaic trough of thirty plates, was passed between the right supra-orbital nerve and the right eye. An immediate, but momentary, improvement of sight was the consequence: the nerves, however, continued to feel the shock till the following day.

June 1st.—The right eye was perfectly clear, and vision increasingly strong.

July.—The cloudiness of the right crystalline was returning, and vision was a little more obscure. The ulceration at the sinciput was merely superficial, and was reduced to scarcely the third of an inch in diameter. It was then extended and made deeper by another application of the pomade.

August.—No opacity of the right crystalline. The sight continues constantly good. The cataract in the left eye had changed from a dead white to a grey colour.

June, 1823.—The right eye is in its natural state, and vision is good. The left crystalline approaches more nearly to a greyish black, but that eye merely perceives the presence of light.

August, 1825.—The cure is permanent with respect to the right eye. The cataract in the left is scarcely visible, but vision on that side has made no progress.

(*Medico-Chirurgical Review*, July.)

### *Observations on the Nature and Importance of the habitual Perspiration of the Feet.*

The importance of healthful perspiration, and its suppression (from whatever cause), are subjects which have often engaged the notice of pathologists. M. Lobstein, however, one of the medical professors at Strasburg, in a paper under the above title, in the Journal Complementaire, has confined his attention to the perspiration of the feet, and has entered considerably at large into its nature,—its influence in health and in disease,—the danger of its sudden suppression, and the most successful method of effecting its re-establishment.

The importance attached to it will appear from the following short account of his paper.

It is stated that the presence of this secretion, in those who are accustomed to it, appears to have great influence over the healthful performance of the various functions of the body; and its incautious suppression frequently gives origin to very obstinate and serious maladies. Our author knows of several individuals, in whom a cessation of this secretion was followed by great mischief.

Sweaty toes despisable
To be found on a tailors shopboard
"You shall nose them in the lobby."

They had previously enjoyed good health; and they did not again regain it until the perspiration was re-established.

M. Lobstein's experience has shown him that the perspiration of the feet is contagious and hereditary. Its contagious nature is proved by its being propagated by wearing the shoes or stockings of a person subject to it; and that it is hereditary, is evinced by its often occurring in all the members of the same family. He has observed, also, that the younger members of such a family have been affected with an unpleasant itching of the skin before the habit of perspiration was firmly established; but, no sooner had they arrived at the age of puberty, and the secretion become manifest, than these affections vanished. The same may be said with regard to certain hypochondriacal and hysterical symptoms; the health of the individual becoming perfectly changed, and remaining good so long as the secretion was properly supplied.

The following are the most common causes of this evacuation being arrested:—Exposure of the inferior extremities to cold or damp; fatigue; cold bathing; as well as the incautious use of astringent applications, such as alum, the oxides of lead, &c. It is affected also by the languid circulation of the lower extremities, incident to old age; and in this case it is sometimes accompanied by a chronic cough, with fetid expectoration. Under these circumstances the secretion will not return, and fatal dropsy usually supervenes.

If imprudently checked, various organs may be affected, and serious, even fatal, maladies induced. Its suppression has sometimes produced apoplexy, melancholy, loss of memory, deafness, toothache, loss of voice, pulmonary consumption, colic, diarrhœa, &c. Rheumatic affections, obstinate ulcers of the feet, with œdema, not unfrequently result.

We may here mention, *en passant*, that our author's observation has led him to remark, that the perspiration of the axillæ is analogous to that of the feet; and that its suppression is attended with similar results.

The next point to be considered, is the best method of restoring the secretion, when improperly or imprudently suppressed. Many modes of practice are mentioned, but the following are the principal:—The pediluvium, which is often of itself sufficient. To render its effect more certain, however, flour of mustard may be added, or some common salt. Another useful remedy is the vapour bath; or a dry bath, composed of hot ashes and warm sand, mixed with salt or malt. The leaves of alders or birch have sometimes been found useful. One other method remains to be noticed: it is wearing stockings made of oil-silk or ox's bladder. Those whose occupations expose them to cold or damp ought always to take proper precautions to preserve the secretion; and this may be done by wearing socks made of wool or felt. In some obstinate cases of suppression, horse-radish or sinapisms to the soles of the feet have been of great service: so also has stick them on when the man's a-dying friction repeated twice a-day, with the application, between the toes, of an ointment composed of equal parts of spirits of hartshorn and mercurial ointment.

It has been questioned, whether or not it be safe to attempt the permanent removal of this unpleasant secretion. This is a subject deserving great attention. It has already been shown that it is of the utmost importance to the health of those with whom it has become habitual, and it has been stated that fatal effects have been produced by its suppression. But it is thought that these occurrences may be averted by a gentle and skilful mode of proceeding, and by carrying off the superabundant humour by some other channel of excretion,—as by the skin generally, by the bladder, or the intestines. With this view, small doses of neutral salts have been exhibited, with the saline mineral waters; and, in addition, the feet may be soaked in an infusion of sage, oak-leaves, rose-petals, and bark.

M. Lobstein does not deny that this plan may succeed in some cases, but he considers it highly injudicious to endeavour to cause a cessation of the perspiration, as it is better to endure the inconvenience, than run the risk of more important evils.

Four cases are given in illustration of the preceding observations: namely, one of violent asthma; another, of a troublesome affection of the stomach; a third, of pulmonary consumption; and a fourth, of violent headache; all of which were clearly deducible from an incautious suppression of this secretion. The third necessarily proved fatal; the other three were cured, so soon as the perspiration of the feet returned. (*Journal Complem.*)

---

### PRACTICAL MEDICINE.

*On the Use of the Oil of Turpentine, in a particular Condition of Fevers.* By Dr. George B. Wood, Professor of Chemistry in the Philadelphia College of Pharmacy.

By those who believe in the specific nature of diseases, and in the specific action of remedies, it has always been considered an object of primary importance to ascertain, by repeated and accurate observation, to what precise symptom, or combination of symptoms, any particular medicinal agent is especially applicable. To a want of such discrimination, it is, I believe, generally attributable, that different physicians meet with such different success in the use of the same remedy in the same complaint; and he who discovers and introduces to the notice of the public a new medicine, or a new application of an old medicine, may frequently blame his own insufficient or inaccurate description of the precise circumstances under which he has found it useful, for the discredit into which it is so liable to fall with the rest of the profession.

The design of the present communication is to point out a certain condition of fever, in which the author believes he has found the spirits of turpentine peculiarly beneficial. This remedy has long been used, with much advantage, in low forms of fever, as a simple stimulant; and it has recently been the fashion to prescribe it in cases accompanied with stomachic or peritoneal inflammation, —in the yellow and puerperal fevers, for example, as a counter-irritant. But, at present, attention is called to it in neither of these capacities.

It is well known to every physician of moderate experience, that, in our protracted remittents, the tongue, instead of cleaning itself gradually from the edges, occasionally throws off the fur at once in large flakes from its surface, which is left red, sore, and of an almost glassy smoothness, totally unlike the natural papillary structure. This appearance will, in almost every instance, be found the harbinger of convalescence, which, however, (as may readily be conceived from the condition in which the mucous lining of the stomach and bowels is left) must be slow, and liable to occasional interruptions.

But sometimes a disposition may be observed in the tongue thus suddenly to throw off its fur; the operation may even have commenced, and proceeded to some extent, so that a considerable patch of smooth denuded surface may be presented, when suddenly the process ceases,—the tongue, if before moist, becomes perfectly dry, and a train of alarming symptoms sets in, which, if not speedily arrested, conduct the patient to almost certain death. I have repeatedly seen instances of the typhus mitior or nervous fever, in which a portion of loosened fur in the middle of the tongue had given me hopes that a favourable crisis was approaching, when the suspension of the clearing process, and the occurrence of a harsh aridity, have given me evidence that the force of disease was overcoming the efforts of nature, and my patient has sunk at the very moment of anticipated recovery. The symptoms which accompany, or immediately succeed, this unpleasant change, are tenderness and slight distention of the abdomen, dark and offensive stools, high-coloured urine, a dry and harsh skin, frequent and feeble pulse, wandering intellect, and an anxious, suffering expression of countenance.

Stimulation with carbonate of ammonia and wine-whey, the use of blisters, of opium, and purgatives, combined sometimes with Serpentaria and Peruvian bark, the malt liquors, and nourishing food,—remedies which my own studies had suggested, or which were recommended by physicians in consultation,—have often proved utterly ineffectual; and the occurrence of tympanitic abdomen, hiccough, and constant low delirium, has proved the immediate precursor of a fatal issue. In one instance, intense abdominal pain occurred toward the close of the disease, and all the symptoms of violent peritoneal inflammation were presented. Examination after death discovered the effusion of the intestinal contents into the cavity of the peritoneum, and one or more large ulcerated openings in the lower extremity of the ileum, plainly indicated the passage through which they had escaped. The inflammation resulting from the presence of this foreign matter had given rise to effusion of coagulable lymph, which covered the external coat of the bowels, and had begun to produce adhesions.

On opening the ileum, its mucous surface was found ulcerated in many places, and the source of the mischief was thus clearly developed. As in this case the occurrence of unpleasant symptoms had been simultaneous with that abortive effort in the tongue, before noticed, to discharge its load, I was led to the supposition that this last appearance might be indicative of the commencement of the ulcerative process in the mucous surface of the intestine. The use of balsams and terebinthinate remedies has proved eminently serviceable in certain states of obstinate chronic diarrhœa and dysentery, in which there was reason to suspect ulceration of the bowels; and an obvious explanation of their operation was, that, by their immediate contact with the ulcerated surface, the morbid action was subdued, and the parts allowed to heal. It required no great exertion of ingenuity to transfer the same reasoning to the cases of fever which I have described, and, on the first opportunity which presented, I prescribed the spirits of turpentine, in the form of julep, in the usual dose of ten or fifteen drops, frequently repeated. The case in which it was given, I had considered almost hopeless; but twenty-four hours had not elapsed before a decided change for the better had occurred, and in a few days the patient was convalescent. The same result has been obtained in other cases of a similar nature, and at present I approach this complication of unpleasant symptoms, not indeed with an absolute certainty of success, but with the confidence which an experience, generally successful, inspires. It is true that I seldom trust the cure to the spirits of turpentine alone, but make use of whatever auxiliary measures the symptoms appear to demand. To this remedy, however, the chief credit is, I think, undoubtedly due; for, before its employment, the best plans of management which had been suggested often proved abortive.

To the imperfectly denuded and arid state of the tongue, which was among the first and most obvious signals of danger, succeeds, by degrees, a new moist brownish or yellowish-white coat, spread evenly over its surface. With this change, an amelioration of the other alarming symptoms is gradually experienced. The tension and soreness of the abdomen are diminished,—the discharges assume a more natural appearance,—the pulse is lessened in frequency, and increased in force,—moisture often covers the skin, —and the mental alienation vanishes. The new fur now slowly begins to disappear, and the patient recovers, as from an ordinary attack of fever.

It cannot be as a stimulant simply that turpentine acts in this form of disease; for the principal danger is obviously not in the prostration of the patient, and the requisite degree of stimulation may readily be obtained from wine or the volatile alkali. In the ordinary prostrated cases of typhus, it often does much good by its powers of exciting and maintaining the vigour of the circulation, and may be used with great advantage in alternation with other medicines of the same class; but, in the particular condition of fever which I have endeavoured to describe, its place can be supplied by no other remedy, and, though the theory of its operation, upon which I ground its employment, may be defective, it must certainly be allowed to exercise, in some way, a specific influence over the disease. (*North American Medical Journal.*)

### *On the Use of Tartar Emetic.*

I had the opportunity of witnessing the administration of tartar emetic in large doses, as a daily medicine, to patients under the care of M. Laennec. His method is to begin with one, two, or four grains, as the total quantity for the twenty-four hours.

A solution is made in the proportion of half or the whole of a grain to half an ounce of simple or some lightly aromatic water, sweetened; and it is given every two hours. The patient is desired to drink very sparingly; for, without this caution, the medicine would most probably produce too much of emetic effect. In the first instance, this result very commonly happens; but it is remarkable how soon the stomach accommodates itself to large doses of this active medicine. When its continued use produces sickness, syrup of white poppy is added, in the proportion of an ounce to half a pint of the antimonial solution; or, as an equivalent, a grain of extract of opium. The total quantity of the tartar emetic is very commonly increased to twenty grains and upwards in the twenty-four hours. I saw an elderly man, who had, in this space of time, on the preceding day, taken sixty grains, without having experienced nausea or any other inconvenience.

M. Laennec considers that the tartar emetic, when administered with the freedom which I have described, exerts a highly useful power in diminishing inflammatory action in continued fever, and in the phlegmasiæ; and he is most satisfied with its action, when, after the first day or two, it ceases to produce any sensible effect on the stomach.

Reflecting on the extraordinary circumstance of the exhibition of this active medicine in such immense quantity with seeming impunity, I thought it probable that the French preparation might be weaker than our own; but, on comparing the crystals which I procured at Paris with those prepared according to the London Pharmacopœia, I could not discover any difference; nor is there the least essential distinction in the mode of preparation, as directed by the London Pharmacopœia and the French Codex. I have lately had many opportunities of prescribing tartar emetic on the principle of treatment which I have described, and I have been perfectly satisfied with its useful agency; but I have usually commenced with one grain, and never exceeded two grains, for the first twenty-four hours; nor found it necessary to go beyond eight in the progressive quantity, except in one case of insanity, in which sixteen grains were given daily for a short time, with the greatest advantage. In the quantity of two grains, it has usually produced considerable sickness for the first day or two; but afterwards even the increased doses have seldom caused any nausea.

With some persons, however, the first dose of a quarter of a grain produces active sickness. It appears to me probable that the maximum of usefulness is to be found in a moderate range of doses, and that it is desirable to avoid trying how much the stomach and the constitution will possibly bear. Have we a security that the accumulation of a very large quantity might not produce violent effects? Indeed, I am informed of an instance in which the amount of sixty grains was taken in divided doses in a short time; the direction being given that the medicine should be repeated till vomiting was produced. At length, such severe sickness did take place, as could not be restrained for many weeks. (Dr. Scudamore's *Work on the Stethoscope, &c.*)

### *Extract of Nux Vomica.*

In a case of long-standing paralysis of one of the lower extremities, I have had great cause to be gratified with the useful agency of the alcoholic extract, in relieving the symptoms of neuralgia. The patient, a gentleman between thirty and forty years of age, had been afflicted with occasional pains of great severity coming on suddenly, causing complete debility, lasting about twelve hours, and during such period producing exquisite tenderness of the limb. With the abatement of pain, sleep followed; and, on awaking, this tenderness had so completely passed away, that he could bear a free handling of the parts; but the muscular power of the limb was weakened during the day,—it was frequently affected with convulsive action, and its usual debility became much increased.

The case is in progress; but up to the present time the medicine has evidently produced good effects, and without causing any tetanic action of the muscles, which I have mentioned as being considered desirable in some cases; although an unusual sense of tightness was produced. Not the least pain has returned, and the limb is stronger. When I had increased the dose to a grain and a quarter during two days, the sense of tightness, joined with much feeling of weight, became troublesome, and I suspended the use of the extract. In forty-eight hours these symptoms disappeared, and the medicine has been resumed without any kind of disagreement. I may add, with satisfaction, that the retentive power of the bladder, which had been for a long time affected, became materially improved. (*Ibid.*)

### STATISTICAL MEDICINE.

### *On Yellow Fever.*

M. le Docteur Andomend recently read a Memoir, entitled "Critical Examination of the Opinions which have prevailed on the Origin of the Yellow Fever." The following are the results:

1st. Was the yellow fever, observed for the first time in 1695 at Martinique, from Siam, as was believed at that time, and from which it got the name of the Siam fever?—The experience of later times proves that the vessels which came from Asia did not communicate that disease, either in America or in Europe.

2d. Does the yellow fever spread itself, in America and in Europe, by means of a *peculiar* virus, like small-pox and syphilis? —This opinion, from which came the first ideas of contagion, has been victoriously opposed by a single consideration, that if the disease proceeded from a peculiar virus, it would be met with in inland countries, where it would remain; whilst it never has been observed but in maritime towns,—that is to say, where there are ships, and where it has come accidentally.

3d. Does the yellow fever depend on the climate of that part of America situated between the tropics; the opinion of Lind?— This is so much the less probable, because this disease has been frequently observed in the United States, and even in Canada; while it has never manifested itself in the seaports of America, or of the Pacific Ocean, although they are within the tropics.

4th. Is yellow fever produced by infection, which exists in seaports of America and Europe; the opinion of M. Devese?—Europe answers this question, that its seaports and its marshes existed before America was discovered, and yet the yellow fever was never seen there till after the discovery of this last continent, and indeed not till 200 years after it. In America, even, it had not been heard of from 1491 to 1695; and the disease has there been so much the more frequent and extended as they have improved the treatment of the Negroes.

5th. Did the yellow fever exist in America before the discovery of that continent; the opinion of M. Moreau de Jomnès?—This opinion is founded on the circumstance that, in times anterior to its discovery, whole villages abandoned certain situations, in consequence of their unhealthiness. The same thing happens in Europe, where we find (particularly in Italy,) villages, formerly flourishing, which have been abandoned because intermittent fevers destroyed the inhabitants: and it is so much the more probable that these same fevers were also fatal to man in America, that it is there the original inhabitants learned to cure them by cinchona.

6th. Has the concourse of Europeans in America been the cause of yellow fever; an opinion of some Spanish physicians?— It is impossible that we should carry into America a disease which we do not experience in Europe. We all there suffer from the effects of climate, and the diseases resulting from it are bilious fevers, which may be taken for yellow fever; an error sometimes committed on both continents.

7th. Is the yellow fever endemic in Guinea; an opinion of Dr. Arrute?—France has officers in Senegal, where they never dreamed of yellow fever. This last opinion is not more founded in reason than the others; but it shows that the author, convinced that the yellow fever of Pont au Passage, in 1823, had come from a vessel which had been in Africa, renounces his belief in the disease originating in America, and that he cannot admit it to have arisen in an European port. Hé was not ignorant that the vessel had been used as a transport; but that did not appear to him so strong as it did to M. Andomend, who has used it as a basis fór some new theories, recently published by him. (*Revue Medicale.*)

---

### SURGERY.

### *Amputation of the Jaw.*

Dr. M'Clellan, professor of surgery in Philadelphia, gives the following account of this operation:—

Mr. Joseph Brown came to this city in the month of July last, from Orange county, New York, with an enormous carcinomatous tumor, which occupied the lower part of his right cheek, and extended over a large portion of the throat on the same side. It commenced on the inferior lip, in the early part of the year 1820, and gradually extended downwards and backwards over the base of the jaw. It was very distinctly circumscribed, and presented a firm cartilaginous kind of structure to the touch. Its margin occupied almost the whole substance of the lower lip, which was drawn very much towards the right by the growth of the tumor in that direction; it then passed downwards across the cheek, over the base of the jaw, just before its angle to the throat; finally, after extending forwards below the jaw, it surmounted the right side of the chin, and terminated near the left commissure of the lips. The lower portion of the tumor which extended into the throat was less prominent than the part above; and, on a careful examination, I ascertained that it was distinctly separate from the original tumor on the face, being made up of secondarily enlarged lymphatic and conglomerate glands. The whole surface of the lip and upper portion of the tumor had been ulcerated for several months previously, from the surface of which a very offensive ichorous matter was constantly discharging. On introducing a probe through the centre of the ulceration, the subjacent bone was found to be carious; which, indeed, might have been suspected from the firm adhesion of the tumor to the side and base of the jaw. Severe lancinating pains had been experienced by the patient for a long period, and his appetite had become very much impaired in consequence of the intolerable fetor of the discharge. His strength, however, and general health, were in other respects very good. There had been no hectic fever; nor had his countenance assumed that shrunken cadaverous appearance, which is so characteristic of a constitutional affection in such cases. The disease had made but very slow progress during the preceding few months, and the pains were by no means increasing in severity. He was only forty-two years old, and felt not a little anxious to protract his life for the benefit of a rising family.

On the 28th of July, I performed an operation for his relief, in the hall of the infirmary of Jefferson Medical College, in presence of the professors and pupils of that institution. An incision was commenced at the left commissure of the lips, and carried downwards, over the symphysis of the jaw and right side of the thyroid cartilage, to the anterior edge of the sterno-mastoid muscle. A second incision was next carried from a little above the right commissure of the lips, around the opposite margin of the tumor, and across the angle of the jaw, until it reached the termination of the former incision. The whole mass of the tumor was then dissected downwards from the surface of the jaw, and from the throat beneath, until all the parts that were included between the two incisions had been removed. By this dissection several enlarged lymphatic glands were removed from the throat, together with the original tumor on the cheek.

After securing the facial and coronary arteries, I next proceeded to examine the condition of the jaw, which was deeply affected with caries. Having satisfied my assistants that it was absolutely necessary to remove it, I divided it by the metacarpal saw in two places,—first, at the symphysis, between the front incisors; and, secondly, a little before the angle, between the second and third molares. On dissecting out the intermediate piece of bone, the facial artery was again divided and secured; after which three more indurated, although not much enlarged, lymphatic glands were removed from the throat. As the surface of the submaxillary gland appeared to be discoloured and tumefied, all that portion of it which lay on the lower surface of the mylo-hyoideus muscle was removed; the remainder, which accompanied the excretory duct above the same muscle, being evidently sound, was left undisturbed. Of course, the facial artery was again laid open by this part of the dissection, and secured, for the third time, much nearer its origin than before. Although the sublingual gland was so fully exposed that it protruded very much, it was not deemed necessary to excise it, on account of its healthy appearance.

The soft parts being now satisfactorily disposed of, I proceeded to examine the posterior extremity of the bone, the cancellated structure of which appeared somewhat discoloured, and bled profusely. As its condition was so suspicious, and as it moreover formed an inconvenient projection, which the integuments could not be made to cover, I applied the saw again just at the angle, and removed about an inch more of the bone, together with the last molar tooth which was implanted in it. The integuments were then approximated, as much as possible, by three interrupted sutures, by means of which the cut extremities of bone were brought much nearer together than before; and the size of the wound also was greatly diminished. Some strips of adhesive plaster, and pledgets of patent lint, secured by a head bandage, completed the dressing; after which the patient was able to walk about with great ease.

In two days after the operation, as the sutures produced a painful degree of tension, and displaced the two portions of bone very much, I removed them, and trusted to the adhesive straps and bandages alone for drawing the opposite sides of the wound together. From that time he complained of no particular inconvenience, and continued to recover very rapidly. In less than ten days the cavity in the throat was entirely closed, and the wound in the face was greatly contracted in size. On the third week he was so nearly recovered from the effects of the operation, that he insisted on returning to his family. The granulations which had sprouted up between the two extremities of bone were then almost cicatrised over in continuity with the integuments, and the deformity was surprisingly diminished.

A letter, dated Orange county, September 2d, has lately been received from Mr. Brown, which states that the parts are now almost entirely healed, and that he has the full power of mastication and articulation.

As it is by no means impossible that there may eventually be a return of the disease in this case, I do not presume to offer a complete history of it in the present communication. The final result will be stated in a paper which I shall hereafter publish, on the probability of affording relief by operations for the removal of specific tumors.

In conclusion, I cannot avoid expressing some degree of surprise at the fact, that intelligent surgeons could ever have anticipated the prevention of hemorrhage, in such operations as I have detailed, by previously securing either of the common carotids. The anastamoses between the branches of all the main arteries of the head are so large and frequent, that I question whether the division of any one of them would not be attended with a troublesome hemorrhage, even after the closure of both carotids. In every case in which the details have as yet been published, it appears that all the divided arterial twigs required the application of a ligature, whether the circulation of blood through their parent trunk had previously been intercepted or not. (*American Medical Review.*)

### *Operation for Imperforate Anus, and Termination of the Rectum in the Vagina.*

In the January Number of "Heckus, Litherorische Annalen der gesammten Heilkunde," for 1826, is a paper by Dr. Dieffenback, a very intelligent and rising physician of Berlin, on Imperforate Anus; in which he recites a case where he performed the operation for the imperforate anus, complicated with cloaca, with perfect success. The patient was a little girl about three months old, well grown for a child of her age, and appeared quite healthy. The external parts of generation were naturally formed; the anus was closed, and the fæces were discharged through a small opening, about one-third of an inch in diameter, in the upper and back part of the vagina. The operation for the removal of this deformity was made at two separate times. At the first, a curved director was introduced into the rectum, through the opening in the vagina, and pressed downwards: a sharp-pointed bistoury was then introduced immediately behind the fossa navicularis, and carried towards the end of the director, but without cutting into it. This incision was extended near to the os coccygis, thus dividing the whole of the perineum; and, when the edges of the wound were separated, the extremity of the rectum could be seen terminating in a cul de sac. The lower part of the gut was then separated carefully with the bistoury from the posterior surface of the vagina, slit open, and allowed to lie in contact with the sides of the first-made wound. The parts were, after the operation, treated with a cold lotion; and, when the suppuration begun, with a tepid fomentation. The opening from the rectum into the vagina, after having been once touched with lapis infunalis, was perfectly closed. For fourteen days, during which time the fæces passed through the newly-made wound, nothing unfavourable occurred, and the edges of the wound, at the end of that time, cicatrised.

In three weeks after the first operation, the second was performed,—namely, for the purpose of forming a new perineum. Drs. Meyer and Gedike, of Berlin, and Messrs. Coulson and Spry, of London, were present. The operation was begun by further separating the anterior surface of the rectum from the vagina, and the sides of the extremity of the rectum, being already adherent to the sides of the former incision: when this anterior part of the gut was separated, it was drawn backwards quite distinctly four or five times by the already adhering parts. The edges of the fore part of the old opening were now cut off, so that they might unite by the adhesive process when brought into contact. The deeper-seated parts were drawn together by a suture, the ends of which were cut off quite close, and the integuments by two small pins, similar to those employed in hare-lip, over which the twisted suture was applied; and thus were the parts effectually secured in contact, and a new perineum formed.

Immediately after the operation, the persons present had the satisfaction of seeing the fæces escape through the artificial anus which remained. A small bougie was introduced into the rectum daily, and the greatest cleanliness directed to be observed. On the fifth day, the suture and needles were removed: a complete union had taken place; and thus was the object of the operation, —namely, the formation of an artificial anus, with the closure of the opening into the vagina, satisfactorily accomplished. (*Med.-Chirurgical Review*, July.)

### MIDWIFERY.

*Case of Rupture of the Linea Alba.* By W. C. Dendy, Esq.

Mrs. Parsons, aged twenty-eight, was delivered of a very large child (her first), after a most severe labour, aggravated too by a premature rupture of the membranes. The pains at the latter stage were for about two hours almost incessant; and, as the presentation was more that of the forehead than of the vertex, I had recourse to the vectis to bring the latter part under the pubes, and finish the labour. After her delivery, I administered the usual anodyne; and on the second evening, a laxative. The contractions at the uterus were regular; and for two days my patient complained of no fixed pain. On the third day, however, I was informed that she had for some hours complained of an acute pain about the umbilicus and pubes, with smarting in the vagina, on the evacuation of urine; which, however, was abundant, as were also the lochia.

There was nothing unusual in these symptoms; but the circumstance which struck me was this: on putting aside the bedclothes, I perceived a large irregular tumor, about midway between the umbilicus and the xiphoid cartilage, at that time about four inches in diameter, and two inches in elevation. Its irregular form at once proved that it was not abscess, and in a few seconds the changes which were continually taking place, both in its shape and size, convinced me that it was a protrusion of some portion of the intestines, whose peristaltic action was now distinctly visible, as if covered but by integument, and which produced the protean alterations alluded to. At some periods, the surface of the abdomen presented its usual appearance; at others, and especially on the lapse of an hour or more, after taking light food, the protrusion would be very considerable, and the peristaltic action visible at some distance from the bed. On the contraction of the recti, I was enabled more minutely to trace the lesion. On passing my fingers along the edges of the canal formed between these muscles, my patient complained of soreness, similar to the friction of a raw surface; and, on pressing more firmly, I was enabled to lay my fingers, as it were, on the cavity of the abdomen. No such surface as that of the linea alba was to be felt affording resistance, and I was convinced that that tendon had given way to a considerable extent.

My first care was to subdue the acute symptoms; and I therefore ordered ten leeches to be applied round the umbilicus, and a blister above the pyramidales. The leeches bled profusely, and the blister rose freely. A cessation of the acute pain was the result. There was still, however, some tension, and a recurrence of the inflammatory symptoms took place on the following morning: the former active mode of treatment was had recourse to, (with the addition of repeated doses of hydr. subm. and pulv. antim.) with the same good effect. It may be mentioned that, during her labour, she felt a snap in her right ear, which was followed by partial loss of hearing; and that, on examination per vaginam, at her request, I discovered that she had slight prolapsus of the uterus.

As the acute symptoms had now subsided, it was proper that I should direct my attention more particularly to the protrusion. I accordingly reduced the then prolapsed viscera, and applied a broad flannel roller round the abdomen over the umbilicus, and as high as the margin of the ribs. The bandage produced an immediate sensation of ease and support to the patient, the previous feeling of debility being directly removed. On the evening, however, soon after the application of the bandage, a discharge of pus, to the amount of about two ounces, issued from the vagina; but from what particular part I could not, from the nurse's account, or from any previous symptoms, determine.

On removing the bandage about a week after its first application, as my patient was lying on her back, I could still feel a considerable depression between the recti, and she complained of a degree of smarting on the pressure of the fingers ; but there was no protrusion. After the lapse of another week, I removed the bandages *as she was sitting.* The hollow was still apparent; but the sensation of soreness much less, and there was no protrusion. It is now a month since the birth of her child; she continues to wear the bandage. The only inconvenience she complains of is from the prolapsus uteri, which, however, is not continual; but is often produced by the contraction of the abdominal muscles, in expelling the intestinal or vesical contents.

I am disposed to believe that, during the excessive pains of labour, very much advantage is derived from compression of the abdomen by bandage; yet such an auxiliary is not generally employed. With a somewhat similar precaution, we support the perineum on the protrusion of the fœtus; and, among persons who are addicted to very laborious exertion, the system of bandaging is by no means uncommon, to preserve a closer compact of muscular fibre, thereby concentrating and increasing power, and of course diminishing the danger of laceration. In modern surgery, too, something like this principle has guided us in the application of adhesive plaster to indolent ulcers, with a view to impart tone and healthy action to those vessels whose office it is to restore the losses, and repair the morbid changes, of the body. (*Med. Repos.*)

---

### MISCELLANEOUS.

*Description of Two Children united together.* By Dr. Berry.

These children are females, three years old this month, (April 4, 1807;) one about thirty-four inches high, the other one-fourth of an inch less. Their features have strong resemblance; the heads of each rather long, and the sides much flattened or elongated by the birth. They are unable to sleep in any other position than face to face. They appear to be connected by their sternums, being united in the same manner as the sides of the letter V. The integuments at the place of joining have the same appearance as the rest of the abdomen, and a ligament can be felt surrounding the junction, which no doubt is the linea alba. When the children are separated from each other, the distance between the top of each sternum is six and a half inches, and between each pubes eight and a half inches, at the full separation. They have only one navel, are healthy, and nowise deformed. Evacuations regular, but at different times. Have had the small-pox at the same time, and favourably. They are daughters of a woman of the Treaver caste. The mother did not suffer particularly in the delivery: the same woman has since had twins, which are living. When they walk, it is sidewise, and something in the form of a circle. If one only is pinched, the other does not feel it; but, if the part uniting their bodies together is pinched, both feel the pain. If medicine is given to one, it affects both. One will awake, while the other will sleep, but both generally sleep at the same time. One is much more lively, and rather stouter than the other. They cross their heads and arms to act or look different ways with ease; they can walk up a stair, and are active when they are playing with other children. One of these children was entirely nourished, for some months after birth, by what it received from the stomach or other part of the alimentary canal of its sister, and still continues to eat rather less: it is, however, as healthy, and nearly of as large a size, as the other.—They were perfect in every other respect, and lived until they were nearly seven years old, when the death of one destroyed the other. Being natives, and at a distance from any medical person, there was no examination after death; nor, perhaps, would the parents have permitted it. (*Transactions of the Medico-Chirurgical Society of Edinburgh*, vol. ii.)

